# Prostate cancer and the influence of dietary factors and supplements: a systematic review

**DOI:** 10.1186/1743-7075-11-30

**Published:** 2014-06-16

**Authors:** Dalvinder Mandair, Roberta Elisa Rossi, Marinos Pericleous, Tara Whyand, Martyn Evan Caplin

**Affiliations:** 1 Centre for Gastroenterology, Royal Free Hospital, Pond Street, London NW3 2QG, UK; 2 Cancer Institute, University College London, Huntley Street, London, UK; 3 Department of Pathophysiology and Organ Transplant, Universita’ degli Studi di Milano and Gastroenterology Unit II, Fondazione IRCCS Ca’ Granda, Ospedale Maggiore Policlinico, Milan, Italy; 4 Department of Nutrition and Dietetics, Royal Free Hospital, London, UK

**Keywords:** Prostate cancer, Nutrition, Diet, Epidemiology, Tumour prevention

## Abstract

**Background:**

Prostate cancer is the second most common cause of cancer worldwide after lung cancer. There is increasing evidence that diet and lifestyle plays a crucial role in prostate cancer biology and tumourigenesis. Prostate cancer itself represents a good model of cancer in which to look for chemopreventive agents due to the high disease prevalence, slowly progressive nature, and long latency period. Dietary agents have gained considerable attention, often receiving much publicity in the media.

**Aim:**

To review the key evidence available for potential chemopreventive nutrients.

**Methods:**

The methodology for this review involved a PubMed search from 1990 to 2013 using the key-words “diet and prostate cancer”, “nutrition and prostate cancer”, “dietary factors and prostate cancer”, “prostate cancer epidemiology”, “prostate cancer prevention”, “prostate cancer progression”.

**Results:**

Red meat, dietary fat and milk intake should be minimised as they appear to increase the risk of prostate cancer. Fruit and vegetables and polyphenols may be preventive in prostate cancer, but further studies are needed to draw more solid conclusions and to clarify their role in patients with an established diagnosis of prostate cancer. Selenium and vitamin supplements cannot be advocated for the prevention of prostate cancer and indeed higher doses may be associated with a worse prognosis. There is no specific evidence regarding benefits of probiotics or prebiotics in prostate cancer.

**Conclusions:**

From the wealth of evidence available, many recommendations can be made although more randomised control trials are required. These need to be carefully designed due to the many confounding factors and heterogeneity of the population.

## Introduction

Despite many advances in the treatment of prostate cancer, little is known about the aetiological factors associated with both its development and progression. It is now the second most common cancer in men worldwide. In 2008, 899,000 men were diagnosed with prostate cancer worldwide, with two thirds of cases seen in the developed world [1]. There is also evidence of increased incidence in immigrant populations to the US and Europe compared to their countries of origin [2]. African American’s have among the highest prostate cancer rates in the world [3], but a study by Chu *et al*. comparing incidence rates across Africa found that although there is considerable variability according to region, prostate cancer rates were still significantly lower than amongst African Americans [4]. There are both hereditary and environmental factors that contribute to prostate carcinogenesis but at present only age, race, and family history are established risk factors [5]. There is increasing evidence from epidemiologic surveys and from laboratory, intervention, and case–control studies that diet and lifestyle plays a crucial role in prostate cancer tumourigenesis. Many nutrients and supplements show potential benefit in helping to slow progression and reduce recurrence, as well as complementing conventional treatment to improve quality of life. Despite the vast amounts of information available, a common consensus on which nutrients may be beneficial and which could be harmful is lacking. This is further complicated by the publication of these articles in the media without appropriate human data available, which can then lead to confusion for both patient and clinician. Here we review the key evidence for the role of different dietary components and nutrients on prostate cancer prevention and progression.

## Methods

A PubMed search was performed for publications from 1990 through 2013, using the following key words, including both medical subject heading (MeSH) terms and free language words/phrases: “diet and prostate cancer”, “nutrition and prostate cancer”, “dietary factors and prostate cancer”, “prostate cancer and epidemiology”, “prostate cancer and prevention”, “prostate cancer and progression”. No language restriction was applied. Reference lists from studies selected by the electronic search were manually searched to identify further relevant reports. Reference lists from all available review articles, primary studies and proceedings of major meetings were also considered. The most relevant nutrients and dietary factors were determined by the level of evidence available, with a minimum level of at least >1 than level IIB evidence as per Scottish intercollegiate guidelines network (SIGN) methodology. The quality and strength level of the results were considered, and we focused the review on meta-analyses and systemic reviews, large epidemiological studies, well designed control or case cohort studies and randomised control trials. If neither of these existed for a nutrient or dietary factor that was frequently purported to have a role in tumourigenesis in other cancers or had received media coverage, then the most relevant *in vivo* and *in vitro* studies were included. Information on clinical trials was sourced from URL: *http://clinicaltrials.gov/*.

## Results

There were a very large number of results returned for each of our search parameters. After filtering for year range, human studies, and article type, the number of articles were as 1058 for “diet and prostate cancer”, 406 for “nutrition and prostate cancer”, 447 for “dietary factors and prostate cancer”, 9965 for “prostate cancer and epidemiology”, 2800 for “prostate cancer and prevention”, and 5743 for “prostate cancer and progression”.

After we manually screened for full text articles and for documents that were specific for the scope of this systematic review, we identified a total of 85 pertinent articles with the strongest level of evidence. In more detail, we considered 14 articles for “meat”, 11 articles for “fatty acids”, 7 articles for “milk intake”, 14 articles for “fruit and vegetables”, 11 articles for “polyphenols”, 21 articles for “selenium and vitamin supplements” and 6 articles for “probiotics and prebiotics”.

### Meat

**Red Meat, Well cooked and Processed Meat** has been suggested to increase the risk of developing many cancers, in particular colorectal and prostate cancer. The mechanisms are thought to involve the generation of Heterocyclic Amines (HCAs) and haem compounds catalysing oxidative damage. HCA’s are produced when creatinine reacts with amino acids and sugars at high temperatures [6]. The longer the meat is cooked and at higher temperatures results in a greater number of HCAs being generated [7]. HCAs have been documented as being some of the most potent mutagens detected using the Ames/salmonella test [8]. Previous *in vivo* studies have demonstrated the increased incidence of breast, colorectal and prostate tumours in animals fed high HCA diets, particularly 2-amino-1-methyl-6-phenylimidazo (4,5-b) pyridine (PhIP) [9]. The greater fat content in red meat compared to white may be an additional factor in promoting carcinogenesis [10].

Many large cohort studies of meat intake have taken place worldwide in the last 20 years with conflicting results. A meta-analysis by Alexander *et al.* looked at prospective studies examining the association between red or processed meat and prostate cancer. Fifteen studies on red meat and 11 on processed meat were included but no association between them and prostate cancer was found [11]. A previous meta-analysis published in 2009 on well-cooked meat and all cancer risk, looked at four studies that examined HCA intake and prostate cancer and concluded there was a positive association with prostate cancer [6].

In a large prospective cohort study conducted at 10 U.S. centers, meat intake, cooking methods and whether cooked till well done was assessed for approximately 29,000 in a National Cancer Institute sponsored cancer screening trial. During the follow-up period, 868 incident prostate cancer cases were identified. A high intake dose–response relation was found for intake of very well-done meat and exposure to PhIP [12]. In another cohort study conducted in the United States, a positive association was also found between high intake of well or very well-done meat and more aggressive prostate cancer [13].

The European Prospective Investigation into Cancer and Nutrition (EPIC)-Heidelberg cohort included a total of 11,928 men aged 40–65 years and 13,612 women aged 35–65 years who were recruited between 1994 and 1998 from Heidelberg and its surrounding communities. Information on diet, lifestyle, and health was obtained at baseline by means of questionnaires and face-to-face interviews. 337 incident cases of prostate cancer (123 advanced cases) were identified among 9,578 men with valid dietary information. Surprisingly there was no association between HCA intake and advanced prostate cancer or between high consumption of strongly browned meat and prostate cancer [14]. This study appears contrary to the previous epidemiological studies, however its findings could be explained in part by genetic polymorphisms that affect metabolism of HCAs, and thus their potential carcinogenic effect [15]. This was suggested in a follow-on case–control study looking again at the EPIC Heidelberg group. All incident cases up to February 2007 were included, which numbered 204 and compared to 360 matched controls. Blood samples were collected for genotyping and they found that the association between HCA intake and prostate cancer risk could be modified by polymorphisms of the genes that encode for HCA metabolizing enzymes such as *GSTT1*, *GSTM1*, and *MnSOD.* Patients with two or more deletions of *GSTT1* and *GSTM1* genes were found to have a higher risk of prostate cancer. Whilst a single nucleotide polymorphism (SNP) in *MnSOD* could also result in a higher risk of prostate cancer due to loss of some if its antioxidant properties [16].

More recently two studies have been published which support the positive association with well done meat and prostate cancer. Di Maso *et al.* analysed data from a network of case–control studies conducted in Italy and Switzerland between 1991 and 2009 (i.e. 1294 prostate cancers) and found that risk of prostate cancer was increased for meat cooked by roasting/grilling [17].

A systematic review by Gathirua-Mwangiet *et al.* suggested that the habitual consumption of a diet high in meat that was well-cooked was associated with an increased risk for advanced prostate cancer, whilst an inconsistent association was observed for intake of total meat [18].

Arab *et al*. analysed the effect of adherence to the World Cancer Research Fund (WCRF) lifestyle recommendations on the risk of highly aggressive prostate cancer in research subjects enrolled in the North Carolina-Louisiana Prostate Cancer Project. This included 2212 newly diagnosed African Americans or Caucasian Americans aged 40–70 years. They reported that consumption of <500 g red meat per week was a statistically significant protective factor in the overall cohort against development of prostate cancer [19].

In light of the available evidence, it is appropriate to suggest limiting intake of well-cooked and processed meat. Red meat should also be limited although the studies are often contradictory, lower intake appears to be associated with reduced risk of prostate cancer. Future studies may benefit from looking for genetic polymorphisms of enzymes that metabolise HCA’s.

Conclusion: well-done meat appears to be associated with an increased risk of prostate cancer and should intake should be restricted. Consumption of <500 g red meat per week may be suggested as a potential chemopreventive strategy.

### Dietary fat

**Dietary Fat** has a role in carcinogenesis but the exact mechanisms are poorly understood.

This observation has been confirmed by Huang *et al*. who investigated the effects of different diets on prostate cancer cell growth using the in vivo and ex vivo model and found that a diet with a high fat intake is associated with increased prostate cancer cell growth. Moreover, they suggested that the serum monocyte chemo-attractant protein-1 (MCP-1) and the CC chemokine receptor 2 (CCRC2) signalling pathway may be involved in prostate cancer progression secondary to high fat diet intake [20].

Different fat subtypes may play a different role in the development of prostate cancer. According to a recent systematic review, total fat intake and in particular, saturated fat intake are significantly associated with an increased risk for advanced prostate cancer, whilst dietary intake of monounsaturated fat, polyunsaturated fat, and linoleic acid is not associated with advanced prostate cancer risk [18].

Persel *et al.* recently suggested that the effect of dietary fat on risk of prostate cancer might differ according to prostate cancer severity. They analysed associations between dietary fats and fatty acids and risk of prostate cancer in the NIH-American Association of Retired Persons (AARP) Diet and Health Study, which included 23,281 prostate cancer incident cases (18,934 non-advanced and 2,930 advanced including 725 fatal cases) among 288,268 men with a median follow-up of nine years. This study showed that saturated fat and α-linoleic acid (ALA) intakes were related to the risk of advanced or fatal prostate cancer but not to non-advanced prostate cancer [21].

The possible association between ALA and aggressive prostate cancer has been suggested by a recent study by Azrad *et al*. who found a positive association between high prostatic ALA levels and more aggressive prostate cancer. They found this was independent of the amount of ALA consumed and suggested this could be due to genetic polymorphism in enzymes related to ALA metabolism [22].

Brasky *et al*. in a large, prospective study found that a high concentration of serum phospholipid long-chain lω-3 polyunsaturated fatty acids (LCω-3PUFA) was associated with statistically significant increases in prostate cancer risk, which suggests a role for these fatty acids in prostate tumourigenesis [23]. These are contradictory to the perception that LCω-3 PUFA’s have many beneficial physiological effects and are considered to be anti-inflammatory [24]. Within the same study they found inconsistent results for ALA and arachidonic acids. These are primary ω-6 fatty acids usually associated with increased inflammation [25]. These findings have been confirmed by a recent case–control study, which showed that long-chain ω-3 PUFA have a relevant role in prostate cancer risk [26].

Phytanic acid is a saturated fatty acid found predominantly in red meats and dairy products. A prospective cohort study looking at the phytanic acid content of foods consumed in the ATBC (Alpha-Tocopherol and Beta Carotene) study found there to be a positive association between high intake and development of prostate cancer [27]. Although small proof of concept studies have found there may be an inhibitory role for a low fat diet on prostate cancer cells, more recent epidemiological studies have not found this to be the case. The European Prospective Investigation into Cancer and Nutrition (EPIC) study included 142500 men and with a median follow up of 8.7 years, 2727 patients were found to have prostate cancer. They found no association between intake of dietary fat and prostate cancer incidence [28].

A recent prospective study analysed 4577 men with non-metastatic prostate cancer in the Health Professionals Follow-up Study (1986–2010) and reported that replacing carbohydrates and animal fat with vegetable fat (heterogeneous mix of monounsaturated and polyunsaturated fats) may reduce the risk of all-cause mortality [29].

Conclusion: High fat intake (mainly saturated fatty acids and linoleic acid) appears to be associated with an increased risk of prostate cancer. The extent of this association may be difficult to interpret due to the heterogeneity in fat subtypes and grade of prostate cancer severity.

### Milk & dairy products

Excessive consumption of milk and dairy products has been postulated to increase the risk of developing prostate cancer. This is thought to be a due to a combination of the fat intake, suppression of circulating 1,25 dihydroxyvitamin D3 which can inhibit cellular proliferation and promote apoptosis and also the increase in insulin-like growth factor-1 from milk containing oestrogen [30]. Recently there was much media coverage of an Icelandic study that looked at milk consumption in 8,894 men born between 1907 and 1935. The study demonstrated that higher daily milk consumption in adolescence (vs. less than daily), but not in midlife or currently was associated with a 3.2-fold risk of advanced prostate cancer (95% CI: 1.25, 8.28) [31]. These striking results confirm what has been suggested in previous epidemiological studies. A meta-analysis of studies published between 1984 and 2003 resulted in a combined odds ratio for milk consumption and prostate cancer of 1.68 [32]. Cell line work has also found milk to stimulate the growth of prostate cancer in culture most recently demonstrated by Tate *et al*. Interestingly, they found that almond milk had a suppressive effect on cancer cell growth [33]. Although no randomised control trials have demonstrated milk exclusion having an impact on incidence, it can be safely inferred from these studies that too much milk and dairy product consumption can increase the risk of prostate cancer and should be avoided. In those patients with established prostate cancer, as yet there is no evidence to recommend that excluding milk and dairy from their diet will have any effect on progression. Indeed, a recent study by Petterson *et al.* found that in a cohort of 3,918 prostate cancer patients, intake was not associated with an increased rate of progression in either early, late or lethal prostate cancer [34].

In their recent prospective cohort study, Song *et al.* confirmed previous findings and also analysed the effects of different types of milk. They showed that higher intake of skimmed/low-fat milk was associated with a greater risk of non-aggressive prostate cancer. Whole-fat milk was consistently associated with higher incidence of fatal prostate cancer and higher prostate cancer-specific mortality among cases [35].

A recent study by Travis *et al.* investigated the hypothesis that a genetic polymorphism in the lactase gene might be associated with elevated dairy product intake and increased prostate cancer risk in a case–control study nested within the EPIC study. The study included 630 men with prostate cancer and 873 matched control participants. They found that lactase genotype frequency varied significantly between countries, with frequencies of the T (lactase persistence) allele ranging from 7% in Greece to 79% in Denmark. The lactase variant was associated with milk intake in men, whilst it was not significantly associated with prostate cancer risk [36].

Conclusion: milk intake, particularly during adolescence appears to be associated with increased risk of prostate cancer and its intake should be minimised. There are no definite data on the effect of milk intake on tumour progression.

### Fruit and vegetables

**Tomatoes and tomato based products** such as ketchup and purees have attracted a lot of interest in prostate cancer. Tomatoes contain lycopene that give them their colour and are one of the main carotenoids consumed in the western diet. It is a powerful antioxidant but may have additional properties in DNA repair that may add to its role as a chemopreventive agent. A meta-analysis in 2004 looked at 21 epidemiological studies related to lycopene and found a weakly protective effect against prostate cancer [37]. Animal studies have shown conflicting results but *in vitro* work has identified pathways where lycopene plays an anti-carcinogenic role. There have been Phase I and Phase II studies that have provided interesting results. A randomised, double-blind, placebo-controlled, one-year study of lycopene 4 mg twice daily was conducted in 40 patients with evidence of HGPIN (high-grade prostatic intraepithelial neoplasia) at transurethral resection of the prostate. The rate of prostate cancer reduction after one year of treatment was 66%. Lycopene was considered an effective chemopreventive agent in the treatment of HGPIN, with no toxicity and good tolerability [38]. The FDA carried out a review of 13 observational studies in 2007 and concluded that at present, there was only limited evidence to support tomatoes and lycopene consumption to decrease the risk of prostate cancer and that larger studies are required [39].

A recent meta-analysis included eleven cohort studies and six nested case–control studies and showed that tomato may play a modest role in the prevention of prostate cancer [40].

Conclusion: Tomatoes and tomato-based products may be preventive in early prostate cancer. Patients with high-grade benign prostate hyperplasia may benefit from increased intake. Further research is needed to determine the type and optimal quantity of tomato products required for prevention against prostate cancer.

**Cruciferous vegetables** are a group of vegetables named for their cross-shaped flowers, and include cabbage, broccoli, brussel sprouts, cauliflower and wasabi. The active components thought to be responsible for their anti-cancer properties are isothiocyanates [41]. They have demonstrated induction of cell cycle arrest, inhibition of tumor invasion and angiogenesis, anti-inflammatory activity and inhibition of extracellular signal-regulated kinases in both *in vitro* and *in vivo studies*[42-44]. Previous epidemiological studies have found some beneficial effects on the prevention of prostate cancer but incorporation of these studies into meta-analyses had not demonstrated a clear benefit. Recently Bosetti *et al.* looked at 7 cohort and 6 population-based case–control studies, and found that a significantly decreased prostate cancer risk was observed overall in the cruciferous vegetables intake group (relative risks = 0.90; 95% confidence interval 0.85–0.96), but not in the subgroup of cohort studies [45].

*Conclusion* ***:*** *cruciferous vegetables may be beneficial but more randomised controlled studies are needed, thus they currently cannot be advocated for prostate cancer prevention.*

**Pomegranate** has been regarded as nature “powerhouse fruit” from the tree Punica Granatum. Its juice contains high concentrations of both tannin and flavonoid anti-oxidants, significantly higher than those seen in green tea and red wine. Both *in vitro* and *in vivo studies* have demonstrated an anti-tumour and anti-proliferative effect. A large multi-centre study performed in 2004 looked at the effect of three preparations of pomegranate supplements on three prostate cancer cell lines and on a xenograft model. This found significant anti-tumour properties of pomegranate mediated by the induction of apoptosis and changes in cell cycle [46]. A role for pomegranate polyphenols in the inhibition of gene expression in androgen receptors in advanced prostate cancer cell models has also been demonstrated [47]. A phase II study recruited 46 patients receiving treatment for prostate cancer whom had rising PSA levels. They were given 8 ounces of pomegranate juice daily and serial PSA levels were taken. They found a significant prolongation of the PSA doubling time from a mean of 15 months to 54 months, along with a prolongation of disease stabilisation [48].

Stenner-Liwen *et al*. conducted a phase IIb, double-blinded, randomised placebo controlled trial in 102 patients with histologically confirmed prostate cancer. Only patients with a PSA value ≥5 ng/ml were included. Participants were given 500 ml of pomegranate juice or 500 ml of placebo beverage every day for a 4 week period, followed by 250 ml of the pomegranate juice daily for another 4 weeks. The study showed that consumption of pomegranate juice as an adjunct intervention in men with advanced prostate cancer did not result in significant PSA declines, compared to placebo [49].

A recent randomised study looked at 70 men awaiting radical prostatectomy who were given either taking either pomegranate extract (POMx) or placebo tablets daily, for up to four weeks before surgery. There were no significant differences in the clinical or pathological features of prostate cancer between each group at the end of the study [50]. The authors did suggest that greater accumulation of pomegranate metabolites within prostate tissue might confer a protective benefit against oxidative DNA damage.

Conclusion: pomegranate supplementation may have a role in both prevention and delaying progression of prostate cancer, but available data are conflicting and the mechanisms involved are poorly understood.

### Polyphenols

**Isoflavones** are a subclass of the flavonoid group, which are polyphenolic substances with strong anti-oxidant properties. Isoflavones occur in plants such as soybeans and other legumes and nuts. The main soy-derived isoflavones are genistein, daidzein, and glycitein. They have weak oestrogen-like properties so are also known as phytoestrogens [51]. Soy intake is high amongst Asian men who have a very low incidence of prostate cancer. This has led to much interest into soy isoflavones as potential chemopreventive agents or even as adjuvants to drug treatment in patients with prostate cancer. *In vitro* studies have found inhibitory effects on signaling pathways, oncogene expression and steroid metabolism [52]. This work has been well supported by *in vivo* work, where a genistein-containing preparation reduced the tumour growth of androgen-sensitive prostatic cancer cells by decreasing proliferation and increasing apoptosis [53]. A review by Messina *et al.* found that in 4 out of 8 studies, daily soy isoflavone intake was associated with a decrease in PSA in patients with prostate cancer [52].

A recent systematic review included eight randomised controlled trials and a meta-analyses of the two studies that only included men with identified risk of prostate cancer found a significant reduction in prostate cancer diagnosis following administration of soy/soy isoflavones. The other six studies included patients already diagnosed with prostate cancer and no significant benefit was identified [54].

A randomised placebo-controlled trial was conducted to examine the effect of soy isoflavone capsules (80 mg/d of total isoflavones, 51 mg/d aglucon units) on serum and tissue biomarkers in patients with localised prostate cancer. Eighty-six men were randomised to treatment with isoflavones or placebo for up to six weeks prior to prostatectomy. Hamilton-Reeves *et al.* showed that genes involved in cell cycle control and in apoptosis were down-regulated in the treatment tumour tissues versus the placebo control, whilst serum hormone levels, total cholesterol, or PSA were not affected by short-term intake of soy-isoflavones [55].

As the age-adjusted incidence rate of prostate cancer has been reported to be lower among Asians than Western populations, a recent Japanese systematic review analysed the association between isoflavones and risk of prostate cancer, focusing on equol, which is converted from daidzein by human intestinal flora and is biologically more active than any other isoflavone **aglycone**. A significant association of isoflavones and of equol-producers with a decreased risk of prostate cancer was found. The authors suggested increasing the proportion of equol-producers by changing the intestinal flora as a possible strategy for reducing the risk of prostate cancer [56].

Conclusion: Soy-containing products may be chemopreventive in prostate cancer but further studies are warranted to clarify their impact on PSA, total testosterone, free testosterone and sex-hormone binding globulin levels in men with, or at risk of, prostate cancer.

**Green Tea** contains high concentrations of polyphenols the most abundant of which is

Epigallocatechin gallate (EGCG). There is much interest in the use of green tea as both a chemopreventive agent and in decreasing progression in cancer. Mechanisms of the anti-tumourigenic action of green tea include apoptosis and cell cycle arrest via alterations in the mitogen-activated protein kinase, phosphatidylinositol-3-kinase (PI3K)/Akt and protein kinase C pathways, inhibition of inflammatory pathways such as cyclooxygenase-2 (COX-2)] and modulation of the insulin-like growth factor (IGF) and androgen receptor axes [57]. Both *in vivo* and *in vitro* studies have provided convincing evidence of the potential benefits of EGCG from green tea. A recent *in vivo* study showed that a combination of green tea and quercetin, a methylation inhibitor, could improve chemoprevention of prostate cancer with no observed side effects [58].

It has been suggested that the lower incidence of prostate cancer in Asian men may be due to the consumption of green tea. A case–control study of 130 patients with histologically confirmed adenocarcinoma of the prostate and 274 controls without prostate cancer or any other malignant disease found the adjusted odds ratio (OR), relative to non-tea drinkers, was 0.28 for tea drinking, 0.12 for those drinking tea over 40 years, 0.09 for those consuming more than 1.5 kg of tea leaves yearly, and 0.27 for those drinking more than 3 cups (1 litre) daily. The study concluded that the risk of prostate cancer declined with increasing frequency, duration and quantity of green tea consumption and that the dose–response relationships were also significant when suggesting that green tea was protective against prostate cancer [59]. There are other much larger epidemiological studies but these have taken place among Asian men and there may be other confounding factors associated with the lower risk of prostate cancer [60]. There have been few randomised controlled trials examining the response of green tea in patients with prostate cancer, whilst those published have yet to demonstrate a clear positive benefit [61].

Conclusion: Supplementation of the diet with Green tea appears to have an effective chemopreventive agent in prostate cancer, but there is no evidence to suggest any beneficial effects in patients already with prostate cancer. There may be benefit in those patients who have HGPIN.

### Selenium and vitamin supplements

**Selenium** is an essential trace element with most intake coming from crop and animal products, but high levels are also found in brazil nuts, tuna, swordfish and molluscs. There has been considerable interest in its antioxidant properties and potential as a chemopreventive agent since a large randomised control trial in the 1990’s found it to be protective against the development of many cancers [62]. This led to many epidemiological studies, 21 of which were included in a meta-analysis by Brinkman *et al*. whom demonstrated an increase in prostate cancer in men with lower selenium levels [63]. Many *in vitro* studies have found Selenium to induce apoptosis, inhibit cellular proliferation and inhibit angiogenesis [64]. Unfortunately, the promising theory behind selenium has not been demonstrated in randomised controlled studies. The Selenium and Vitamin E Cancer Prevention Trial (SELECT) found no reduction in risk of prostate cancer with either selenium or vitamin E supplements. This large study randomised 35,533 men from 427 study sites in the United States, Canada, and Puerto Rico to either selenium vs placebo, vitamin E vs placebo or both vs placebo. Worryingly, an elevated risk with vitamin E was found [65]. A possible explanation for these null findings include the agent formulation and dose, the baseline characteristics of the cohort and the study design, which suggests that it is likely that only specific subpopulations may benefit from selenium supplementation [66]. Furthermore, a recent phase III studies of selenium vs placebo in patients with HGPIN found no benefit in the prevention of progression to prostate cancer and suggested higher intake might increase the risk of cancer [67]. There are of course confounding factors that may influence the outcome of such studies such as selenium status and genetic polymorphism of enzymes involved in selenium metabolism [68]. A comprehensive review by Venkastweran *et al*. of chemoprevention in prostate cancer confirmed our conclusion that despite encouraging *in vivo* and *in vitro* data, epidemiological studies and the larger case–control studies had demonstrated a need for caution in advocating selenium supplementation until there is improved understanding of its metabolism [69].

*Conclusion: large randomised trials showed no role for selenium supplementation in chemoprevention of prostate cancer and emerging evidence suggests that high levels may be pro-carcinogenic*.

**Vitamin E** is a general term used to refer to a group of naturally occurring compounds called tocopherols and tocotrienols, as well as vitamin E derivatives such asacetate, succinate, and nicotinate of both natural and synthetic alpha tocopherol [70]. It’s strong antioxidant and inhibitory properties were postulated to have a chemopreventive role in the development of many cancers. The Alpha-Tocopherol and Beta-Carotene (ATBC) cancer study in 1994 demonstrated a one-third reduction in prostate cancer incidence and a 41% reduction in prostate cancer mortality among Finnish male smokers receiving Vitamin E. No significant difference was seen in non-smokers given Vitamin E and no beneficial effects were seen with Vitamin A [71]. *In vitro* studies have followed, reporting encouraging results such as a 40% drop in the number of cancer cells with 72 hr treatment with vitamin E succinate [72]. In a study by Venkastweran *et al*., an animal model of prostate cancer using lady transgenic mice found a significant reduction in incidence of prostate cancer where mice were given vitamin E supplementation [73]. A further study by the same group found that mice given supplementation at an early stage with Vitamin E, Selenium and Lycopene had only a 10% incidence of prostate cancer compared to 75% in the control group. A combination of Vitamin E and Selenium was less effective but still resulted in decreased incidence and improved overall survival in the mice [74]. Unfortunately, this encouraging data has not been seen in human studies and the SELECT study found that a higher incidence of prostate cancer was seen in patients given vitamin E supplementation [65].

Recently, Bauer *et al.* examined circulating vitamin E and vitamin E-related genes in relation to the risk of high-grade prostate cancer and prostate cancer recurrence among men diagnosed with organ-confined disease (35,533 men from the US, Canada, and Puerto Rico were enrolled in a 3-year period). They found that germ line genetic variation in genes of enzymes associated with detoxification of free radicals may be associated with the risk of high-grade disease at diagnosis and disease recurrence, which might suggest consideration of such genotypes in the interpretation of vitamin E supplementation trials such as SELECT. Additionally, circulating γ-tocopherol levels may also be associated with an increased risk of high-grade disease at diagnosis [75].

Conclusion: Vitamin E supplementation should not be recommended as chemoprevention in prostate cancer at present, until better understanding of its biology is available.

**Vitamin A** consists of Retinol and the carotenes; alpha-carotene, beta-carotene, gamma-carotene, and the xanthophyll beta-cryptoxanthin. Induction of apoptosis by retinoids has been observed in various prostate cancer cells *in vitro* and *in vivo*[76]. However, large randomised control trials have not found any protective benefits and some studies have suggested an increase in the incidence of various cancers with the use of multivitamins. The most significant was the Carotene and Retinol Efficacy Trial (CARET), a multicentre randomised, double-blind placebo-controlled chemoprevention trial testing whether daily supplementation with 30 mg β-carotene + 25,000 IU retinyl palmitate would reduce lung cancer risk among 18,314 heavy smokers, former heavy smokers and asbestos-exposed workers. Another arm of the study was to look at prostate cancer. Men in the interventional arm who also used additional dietary supplements had an increased RR towards developing an aggressive prostate cancer (RR 1.52, 95% CI 1.03–2.24; *P* < 0.05) [77]. Although this study included only smokers or former smokers, similar studies have suggested that vitamin A and multivitamin supplements may increase the risk of prostate cancer.

Conclusion: Vitamin A supplements are not recommended as part of chemopreventive diet to prevent prostate cancer.

**Vitamin D** There has been conflicting evidence about the effect of Vitamin D on prostate Cancer. *In vitro* studies suggest it may have a useful role in all types of cancer in view of its antiproliferative properties and potential to induce apoptosis. Dietary consumption of Vitamin D can have a significant effect on circulating levels of both metabolites 25(OH)D and 1,25(OH)2D and both metabolites may have a chemopreventive potential [78]. Many pre-clinical studies have found that murine models of prostate cancer with vitamin D deficiency show an increased rate of proliferation and of growth in bone [79]. Epidemiological studies have suggested there may be even be a U-shaped relationship with vitamin D and prostate cancer, with very low levels or very high levels associated with increased risk. A study by Ahn *et al.* detected a significant linear increase in the risk of aggressive prostate cancer for those with 25(OH) D concentrations higher than 42 nmol/L [80]. Much of the evidence is based upon epidemiological studies, and although a U-shaped curve may be apparent, more recent evidence suggests that Vitamin D has neither a protective or causative role in prostate cancer or in the development of metastases. A large meta-analysis by Gilbert *et al.* in 2010 looked at 25 papers from a database of over 24,000. It found there to be no published literature that supported a strong role for high or low vitamin D levels in either preventing prostate cancer or its progression [81]. Holt *et al.* conducted a population-based cohort study of 1476 prostate cancer patients to assess disease recurrence/progression and prostate cancer-specific mortality risks associated with serum levels of 25(OH)D. During an average of 10.8 years of follow-up, they found that serum levels of 25(OH) D were not associated with risk of recurrence/progression or mortality from prostate cancer [82]. A recent meta-analysis, including thirty-four studies for a total of 10,267 cases and 11,489 controls, focused on the association between vitamin D receptor (VDR) polymorphisms, which mediate the cellular effects of vitamin D and risk of prostate cancer. No evidence to support an association between any of the VDR polymorphisms and risk of prostate cancer was found [83]. Further studies have found there to be no convincing association and random control trials are lacking.

Conclusion: Vitamin D supplementation in prostate cancer cannot be advocated unless the patient is deficient, and even then must be replaced with care as higher doses may be associated with a worse prognosis.

### Prebiotics and probiotics

**Prebiotics are** defined as “non-digestible food ingredients that beneficially affect the host by selectively stimulating the growth and/or the activity of one or a limited number of bacteria in the colon” [84]. They are fermented by bacteria, benefiting the host by restoring appropriate microbial balance in the intestine. There are two main groups of prebiotics; Oligosaccharides which are fructooligosaccharides (FOS) and inulin, and maltooligosaccharides of which maldrexitin is most researched [85]. Inulin-type fructans are present in foods such as garlic, onion, artichoke and asparagus. There is little data looking for chemopreventive use in prostate cancer, whereas benefit has been proposed in other cancers, e.g. colorectal cancer [86]. Possible mechanisms for a protective role in carcinogenesis include: (i) selectively promoting the growth of bacteria such as bifidobacteria which have a tumour suppressive effect (ii) the formation of reducing agents, such as glutathione which can inactivate food-borne carcinogens (iii) decreasing levels of certain bacterial enzymes purported to be involved in activation of carcinogens (iv) the production of anti-cancer metabolites such as short chain fatty acids (SCFA), in particular butyrate (v) the up-regulation of apoptosis (vi) causing a decrease in triglycerides, phospholipids and low-density lipoproteins, which are required for tumour growth [87].

**Probiotics** are “live microorganisms, which, when administered in adequate amounts, confer a health benefit on the host” [88]. Probiotics have been shown to have effects on irritable bowel, metabolic syndromes and urogenital infections, and as potential chemopreventive agents in colorectal cancer [86]. Possible mechanisms in chemoprevention are similar to those of prebiotics that promote a healthy microbial balance in the intestine. They are also thought to have additional roles in decreasing expression of cytokines and upregulation of intestinal genes that are involved in nutrient absorption, mucosal barrier fortification, xenobiotic metabolism, and angiogenesis [89]. There have been very few studies looking at a potential chemopreventive role in prostate cancer, although there are many human studies in colorectal cancer and some animal studies in breast cancer.

Conclusion: Currently, although there are potential general health benefits from supplementation with pre- and probiotics, there is no direct evidence regarding benefits in prostate cancer.

## Conclusion

There is a wealth of scientific information available on the potential role of diet in chemoprevention in prostate cancer. Much of the data is encouraging and advocates an important role for optimising intake of a wide variety of nutrients. Herein, we analysed nutritional factors that might play a role in the development of prostate cancer (Table 1). There is evidence that consumption of red meat, dietary fat and milk intake should be minimised, as they appear to increase the risk of prostate cancer. Fruit and vegetables and polyphenols (i.e. green tea and soy-products) may be preventive in prostate cancer, but further studies are needed to draw more solid conclusions and to clarify their role in patients with an established diagnosis of prostate cancer. Selenium and vitamin supplements cannot be advocated for the prevention of prostate cancer and indeed higher doses may be associated with a worse prognosis. There is no specific evidence regarding benefits of probiotics or prebiotics in prostate cancer, thus they cannot be advocated in this setting.

**Table 1:** Summary of current evidence on the relationship between dietary factors and supplements and risk of prostate cancer

1.	Well-done meat is associated with increased risk of prostate cancer; consumption of red meat should be limited to <500 g per week.
2.	High fat intake (mainly saturated fatty acids and linoleic acid) appears related to increased risk of prostate cancer.
3.	Milk intake appears to be associated with increased risk of prostate cancer and its intake should be minimized.
4.	Tomatoes and tomato-based products may be preventive in early prostate cancer.
5.	Cruciferous vegetables may be beneficial but they currently cannot be advocated for prostate cancer prevention due to the paucity of randomized trials.
6.	Pomegranate may have a role in both prevention and delaying progression of prostate cancer, but available data are often conflicting.
7.	Soy-containing products may be chemopreventive in prostate cancer but further studies are warranted to clarify their impact on PSA, testosterone, and sex-hormone binding globulin levels in men with, or at risk of, prostate cancer.
8.	Green tea appears a chemopreventive agent in prostate cancer, but there is inconclusive benefit in patients already with prostate cancer.
9.	Selenium supplementation is not recommended in chemoprevention of prostate cancer and very high levels may indeed be pro-carcinogenic.
10.	Vitamin A is not recommended as part of chemopreventive diet to prevent prostate cancer.
11.	Supplementation with vitamin D is not advocated unless the patient is vitamin D deficient. High levels of vitamin D may be associated with a worse prognosis.
12.	There is no evidence regarding benefits of pre- or probiotics in prostate cancer.

There is a need for larger randomised control trials, which will also help to identify optimal dose and duration of nutrients as well as potentially determine why some sub-groups of patients may respond better than others. However, heterogeneous populations with differing lifestyles, baseline nutritional characteristics, genetic background and many other variables mean it is difficult to design such studies in order to develop population-based prevention strategies for prostate cancer.

## Competing interests

The authors declare that they have no competing interests.

## Authors’ contribution

All authors contributed to the writing of this manuscript.
